# “I Just Feel Disconnected”: How Feelings of Shame Relate To Experiences of Trauma in People With Psychosis

**DOI:** 10.1007/s10597-025-01525-1

**Published:** 2025-10-02

**Authors:** Kimberley Davies, Sophie Isobel, Zachary Steel, Sarah Morgan, Julia M. Lappin

**Affiliations:** 1 Discipline of Psychiatry and Mental Health, School of Clinical Medicine, Faculty of Medicine and Health, UNSW Sydney, Kensington, NSW 2052 Australia; 2 The Tertiary Referral Service for Psychosis, Prince of Wales Hospital, Randwick, NSW 2031 Australia; 3 Faculty of Medicine and Health, The University of Sydney, Camperdown, NSW 2006 Australia; 4 Early Psychosis Program, South Eastern Sydney Local Health District, Sydney, NSW Australia

**Keywords:** Psychosis, Qualitative, Shame, Trauma, Schizophrenia

## Abstract

Research suggests shame plays a mediatory role in the relationship between trauma and psychosis, however, there is limited information on how this may occur. This qualitative study explored how experiences of shame in people living with psychosis is linked to past trauma. Fourteen adults completed semi-structured interviews. Reflexive thematic analysis was used to develop four themes: (1) shame maintains trauma; (2) trauma-related shame drives disconnection from the self; (3) avoiding shame from trauma leads to disconnection from aliveness; and (4) pervasive shame from trauma leads to isolation. The findings suggest that shame may play a key role in maintaining the influence of trauma on sense of self and on the way people engage with the world, including in the context of psychosis. Future research could explore whether addressing shame through intervention has any impact on the influence trauma has on people experiencing psychosis.

**Supplementary Information:**

The online version contains supplementary material available at 10.1007/s10597-025-01525-1.

## Introduction

Psychological factors play a central role in understanding the onset and course of experiences of psychosis (Garety et al., 2001). Seminal psychological models have focused on the interaction between cognitive and affective processes thought to contribute to psychosis. For instance, emotional distress arising from anxiety and mood-related difficulties is considered to shape the development of appraisal frameworks, which in turn influence the meaning ascribed to anomalous experiences (Fisher et al., 2013; Garety et al., 2001). More recently, a range of broader emotions, including ‘social emotions’ such as shame and loneliness have been proposed as also important for understanding the experience of psychosis (Heriot-Maitland et al., 2022).

Shame is often described as a self-conscious emotion, arising from appraisal that consequences of an event are due to personal failure, and that the aspects of the self contributing to this are stable and uncontrollable (Tracy & Robins, 2006). This can lead to evaluations of the whole self as being bad or defective, and cause significant distress (Gilbert, 2003). The experience of shame elicits feelings of being exposed, often leading to social withdrawal and avoidant behaviours (Tangney, 1993). From an evolutionary perspective, shame serves an adaptive function in alerting an individual to the potential of being negatively evaluated by others, and in turn, the threat of being socially excluded or rejected (Gilbert, 2003). However, ongoing shame has been linked to a range of mental health difficulties including depression (Kim et al., 2011), anxiety (Cândea & Szentagotai-Tătar, 2018) and psychosis (Davies et al., 2024). Shame following adverse events has also been linked to poorer psychological outcomes such as the development of PTSD (La Bash & Papa, 2014).

There are many potential sources of shame for people living with psychosis, including parts of experiences of psychosis themselves, for example, hearing derogatory voices. Previous work has found that people living with psychosis experience shame associated with their behaviours whilst unwell, in addition to the secondary impacts of psychosis, such as missed life opportunities and experiences of mental health care (Davies et al., 2025a). Experiences of psychosis can also exacerbate existing shame, with shame from past adverse experiences being reactivated by psychosis (Davies et al., 2025a).

Adverse life events are reported in higher rates in people with psychosis than the general population (Varese et al., 2012) and exposure to earlier trauma, particularly in childhood has been identified as a key factor in the later development of psychosis experiences (McGrath et al., 2017). A recent study suggests shame may play a mediatory role in this relationship (Davies et al., 2025b). One potential path through which this could occur, is via shame’s influence on coping responses, specifically the adoption of absorbed and avoidant strategies such as rumination, hypervigilance, emotional suppression and dissociation (McCarthy-Jones, 2017). Additionally, models of distress such as the Power, Threat, Meaning Framework (PTMF), identify that shame may shape the meaning people make of the experiences and subsequently influence the way they manage distress (Johnstone et al., 2018). However, there is currently limited information on how experiences of shame in people living with psychosis are linked to past trauma.

This study aims to address this gap through conducting an interpretive qualitative analysis of transcripts collected as part of a broader qualitative inquiry into the role of shame in psychosis experiences. Initially, data were analysed to explore experiences of shame from the perspectives of people living with psychosis (Davies et al., 2025a). While the focus was initially on shame and psychosis, experiences of trauma were apparent throughout the participant accounts. Thus, to build on these findings and contribute to understanding the nature of the relationship between shame, psychosis and trauma, this study was undertaken to explore how experiences of shame in people living with psychosis relate to trauma.

## Methods

An interpretive qualitative inquiry was undertaken guided by the research question ‘How do feelings of shame in people with psychosis relate to past experiences of trauma?’. Interpretive inquiry seeks to go beyond the ‘what’ in qualitative data and understand the ‘how’ (Wiesner, 2022), contextualising the data within broader theory and frameworks. The analysis was part of a wider body of work which had identified a measurable relationship between shame and psychosis (Davies et al., 2024) and explored people’s understanding of this relationship (Davies et al., 2025a). This part of the work sought to more deeply understand the role that trauma played in this relationship, drawing on the participant data and ideas of ‘value-adding analysis’ (Eakin & Gladstone, 2020). Value-adding analysis requires moving beyond thematically concepts to theorising how concepts may relate to each other in a more abstract way. The 32-item Consolidated Criteria for Reporting Qualitative Research (COREQ) checklist was used to guide study reporting (Tong et al., 2007).

### Setting

Interviews were conducted between July 2023 and June 2024 across three mental health services in Sydney, Australia including: an adult community mental health service; an adult mental health rehabilitation inpatient service; and a youth focused community based early intervention for psychosis service. The study received ethical approval from [South Eastern Sydney Local Health District] HREC (Ref: 2022/ETH02000).

### Participants

Participants engaged with the three services were invited to participate if they: had a lived experience of psychosis; were aged 18 years or older; and were able to provide consent. Mental health clinicians within the included services identified individuals who met criteria and made initial contact. Individuals who expressed interest in the study to the clinician, and agreed to be contacted by the study team, were followed up by (KD) to discuss the study procedures in further detail and were provided with the study information. Basic demographic data were collected to ensure a diverse sample. The sample reported here were aged between 21 and 64 years and the majority identified as female (86%), 71% reported their cultural identity as Anglo-Australian and had an illness duration ranging from 1 to 43 years. All participants reported experiencing interpersonal trauma including bullying, domestic violence, physical and sexual abuse. Participants were provided with a gift card (AUD $100) for taking part in the study in recognition of the time that participation required.

### Data Collection

Participant experiences were explored through semi-structured interviews. This format allowed individual perspectives to be explored in-depth, whilst providing some structure to the discussion through a topic framework which was applied across all interviews (Kallio et al., 2016). This flexible approach promoted a conversational tone and allowed participants to guide the interview through opportunities to raise issues meaningful to them. This was important given the sensitive nature of the interviews.

The interview guide focused on three broad areas: participant’s experiences of psychosis; experiences of shame, experiences of adverse life events; and experiences accessing mental health services. Interview questions focused on participants’ reflections of their experiences, emotions that arose at the time and in the present (including shame), and the meanings participants made of their experiences. The guide provided an overarching structure for the interviews, however, the order of questions and depth of discussion varied according to participant engagement and natural flow of conversation. An initial review of the interview questions was undertaken by individuals with lived experience of psychosis and clinicians with expertise in supporting individuals with experiences of psychosis, to assess language and clarity (Kallio et al., 2016). Ongoing review of the interview questions was undertaken and changes made where appropriate. For instance, wording and ordering of questions were adjusted in response to participant feedback and emergent findings (see supplementary material).

A trauma-informed approach informed both the design and conduct of interviews (Isobel, 2021b). For example, the use of language was carefully considered in the development of the interview questions and checked for acceptability to participants. Where participants preferred alternate terms, including for ‘psychosis’, these were adopted throughout the interview. Participants’ sense of safety was prioritised throughout the study process and procedures were adapted when needed. This included offering participants a copy of the interview questions prior to the interview and attempts to minimise causing or triggering shame throughout the study where possible. Actions to manage this included frequent reflection and discussion amongst the research team, observing participant cues and feedback throughout data collection and ensuring any quotes used in reporting would not identify participants or evoke feelings of exposure.

Fourteen participants completed an interview. All interviews were conducted by the first author (KD), on-site, at a time chosen by the participant. The interviewer completed a number of practice interviews with volunteers under supervision prior to commencing participant interviews. After each of the first three interviews the audio-recording and transcript were shared with the research team to provide further feedback. Interviews ranged between 27 and 77 min (average 48 min) and were audio-recorded and transcribed. Data were continually reviewed throughout the interview process, and data collection ceased when considered to be rich and incorporating a range of experiences in depth to sufficiently answer the research question (Braun & Clarke, 2021). A summary of the interview transcript was offered to all participants to allow for review and feedback. Six participants requested a transcript summary and confirmed their agreement with the contents, no changes were proposed.

### Data Analysis

The focus of the analysis was on interpreting the relationship between trauma, shame and psychosis based on the experiences of people living with psychosis. Analysis was guided by reflexive thematic analysis (Braun & Clarke, 2022). The themes were developed by the researchers based on how participants talked about shame and the links apparent between trauma and shame. Interpretation is inherent in all qualitative research and refers to credible sense-making by researchers, based on immersion in descriptive material (Gilgun, 2015). Interpretation is a crucial component of thematic analysis and is inherently reflexive and requires trustworthiness of process, as well as creativity to create ‘working hypotheses’ of how concepts interrelate (Gilgun, 2015).

Braun & Clarke’s six phases of thematic analysis were followed (Braun & Clarke, 2022). An initial review of each transcript alongside the audio-recoding was conducted to allow familiarisation with the data, before each transcript was revisited and notated with early thoughts and ideas. Data were coded in NVivo (Version 12, QSR International Pty Ltd., 2018) in response to the research question. The coding process was iterative with codes arising from more recent transcripts applied to those previously coded. Codes were then grouped into candidate themes that reflected broad concepts in the data such as those that described the ways participants managed shame. Guided by the research question, interpretive themes were developed from across the data that described possible mechanisms of the relationship between key concepts. Draft themes were discussed and refined amongst the research team, in the context of existing literature and research, and in consideration of ‘telling a story in a way that makes sense of’ (Braun & Clarke, 2022, p. 197) the nature of the relationship between trauma, shame and psychosis. Alongside a student researcher, the research team consisted of a psychiatrist, clinical psychologist, mental health nurse and social worker, all with significant experience supporting individuals experiencing psychosis and trauma and each bringing clinical expertise to the team discussion. Each interpretive theme is described, named using participants’ words and illustrative quotes included to add depth (Lingard, 2019).

## Results

Across the interviews participants shared their experiences of shame, and the meanings associated with them. Exploration of how these experiences may relate to past trauma suggests shame arising from trauma plays an active role in driving disconnection from the self and others. Shame can also influence the relationship between trauma and experiences of psychosis. The themes across the data were: shame maintains trauma; trauma-related shame drives disconnection from the self; avoiding shame from trauma leads to disconnection from aliveness; and pervasive shame from trauma leads to isolation.

### Theme 1: “Everyone’s a Stripe and I’m a Spot”: Shame Maintains Trauma

The ways participants described trauma suggested that shame can contribute to the sustaining impacts of trauma. While traumatic events occurred in the past, shame was predominant in how trauma continued to impact in the everyday. This was apparent in the links participants drew between traumatic experiences and the ways they perceive themselves. Manifestations of shame were often linked to these experiences, such as feeling damaged or less than others. These feelings were then woven through their descriptions of their perceptions of ‘self’ over time. For example, during traumatic experiences, they may feel worthless or inferior, which then became a pervasive perception of themselves throughout new interactions in their life. Participants could recognise how these understandings of self arose from trauma, for example:

*“I think when I was being bullied*,* I felt like I was very small and insignificant. And I’d often try and make myself small as well*,* instead of taking on space.”* – P14.

There were also descriptions of shame linked more directly to recent incidents and events, including their experiences of psychosis. While feelings of shame from past trauma were reflected in the ways participants described themselves and how they interact with the world, when reflecting on shame associated with recent events, participants focused more on how they may be perceived or judged by others. For example, participants reflected on pervasive feelings of separateness from others or a sense of there being ‘something wrong with them’. Psychosis could then ‘confirm’ these feelings of wrongness or difference, reinforcing earlier trauma-related beliefs such as:

*“I feel like I’m not an equal. I’m not even with everyone else. Everyone’s a stripe and I’m a spot … That’s how I generally feel to this day.” – P2*.

Shame seemingly continued to shape the way people made sense of more recent difficult experiences. For example, participants described how they would assume fault for situations out of their control or that they felt undeserving of positive life opportunities. Shame linked to past trauma, remained a lens through which adversities were understood, and could also be acutely re-activated in the present, through reminiscent experiences of powerlessness, including those experienced in mental health care.

*“…I was like*,* “I’m not good enough*,* am I not good enough”. Like this internal battle of I don’t really feel like I fit in … This internal struggle of do I belong here.”* – P13.

Participants also described ways in which they were hypervigilant to potential sources of shame or situations that may provoke shame associated with past trauma, recognising that trauma related triggers could activate negative perceptions of self. For example, one participant drew links between trauma-related memories from the past and feelings of being unclean in the present:

*“There’s this guy …he triggers me…. so I avoid him and every time I see him I’m like… I need a shower” – P7*.

### Theme 2: “I Don’t Know Who I Am, I dDon’t Know Who I Am”: Trauma-Related Shame Drives Disconnection From the Self

The accounts showed various ways in which trauma through the experience of ongoing shame could lead people to disconnect from themselves. Various ways that this occurred were apparent. For example, participants shared situations where they would minimise or deny their own needs to keep the peace or because they did not feel important enough. Participants could draw links between a loss of self and patterns of behaviour over time, for example:

*“I looked for ways to get approval from people*,* especially later on [in] romantic relationships… I would just go along with things that I didn’t want*,* I didn’t check in with myself. Is this okay for me? I just was like*,* if this person wants it*,* then I’ll do it.” – P14*.

Participant experiences highlighted how disconnecting from the self could occur in a way that was beneath consciousness, as a means of escaping the distressing effects of shame arising from past trauma. For example, participants seemed to disown aspects of themselves they considered damaged, or that had been associated with past rejection. Participants spoke of these parts in ways that separated them from their core self or associated shame with them. This was reflected in accounts of avoiding things that reminded them of their younger selves due to shame about core parts of self. For some this led to feeling a disconnection from their whole identity and a lack of a sense of who they are. For example:

*“And then I became very*,* very depressed because…I didn’t know who I was…I kept on saying to myself*,* “I don’t know who I am*,* I don’t know who I am.” - P12*.

This experience was identified as being directly linked to past trauma and yet feelings of disconnection became exacerbated by psychosis experiences when people felt unsure about who they were or whether they could trust themselves. A deep sense of instability for many participants describing how they doubted their own capacity to know themselves.

### Theme 3: “I’ll Just Squash It Under the Rug”: Avoiding Shame From Trauma Leads to Disconnection From Aliveness

Participants identified that negative emotions, arising from trauma were shameful and needed to be avoided, which over time led to a sustained pattern of emotional suppression, feelings of numbness and emotional denial. Throughout the interviews, this was apparent when participants were not able or willing to engage in discussions about negative emotions. Some participants would use the third person voice when discussing adverse or traumatic events or would only discuss such events in relation to other people rather than themselves. While a disconnection was apparent in how some participants talked about trauma, others were explicitly aware of this technique, for example:

*“ … I’ve been through all these things but I haven’t really connected or attached to these things properly. The events happen and I just go in my head*,* how can I move forward and overcome this*,* and then I’ll find a way to overcome it and just disconnect.” – P11“*.

When numbness or suppression wasn’t effective, participants may use distraction strategies to avoid experiencing emotions or difficult thoughts, for example.

*“If I’m ever tempted to think about things that aren’t relaxing*,* I’ll bury my head in my physics and feel better.”* – P9.

When reflecting on their understandings of shame, participants described experiencing a kind of anticipatory shame which led them to avoid thinking about or remembering past traumatic events, and at times avoiding anything related to their past out of uncertainty of what emotions or thoughts could be activated.

However, rather than this reducing shame, this disconnection could paradoxically enhance shame, leading people to not share their experiences with others, avoid all emotions even joyful ones, or not seek support out of fear. Participants described feeling ‘nothing’ or ‘numb’ and struggled to connect events and emotions, leading the past and the present to seem blurred, with participants confused about what they were thinking or feeling currently or historically, and why.

*“I feel pretty numb I reckon with emotions. Maybe a little sadness*,* but I’ve definitely fluctuated with hearing terrible voices saying I’m a piece of shit but then I’d go to laughing five seconds later*,* uncontrollable giggling kind of. So I don’t really know. It’s a really confusing state to be in but generally [I’m] probably just numb.” – P10*.

Blocking out, or avoiding thinking about, past experiences could lead participants to consider that they experience psychosis because of being inherently damaged, rather than recognising any link between trauma and mental health experiences. This could lead to a deepening of internalised shame.

### Theme 4: “I Had Difficulty Looking People in the Eyes”: Pervasive Shame From Trauma Leads to Isolation

Participants’ demonstrated disconnection from others occurring to manage shame and protect themselves from further shame. Shame could lead people to expect shame within social interactions, leading to a cycle of avoidance. Participants described avoidance of social connections to minimise shame, and then further shame about their subsequent isolation and separateness. For example, when discussing shame directly, one participant described:

*“it makes it very hard for me to get out of my shell. I’ve been a loner most of my life*,* and when I socialise*,* I seem to get humiliated or don’t fit in”* – P2.

Withdrawal from others could occur in anticipation of shame associated with people viewing their undesirable parts of their self, or from feeling unworthy. In this way, even when people did connect with others, they could still be preoccupied with hiding parts of themselves or feeling disconnected. As one person described:

“*I feel like I’m less than human*” - P2.

Experiences of isolation were amplified by psychosis and mental health care. Participants described and implied ways that their psychosis could confirm their differentness, positioning psychosis as consequential to parts of themselves that they try hard to hide. Experiences of care could lead to a loss of control and humiliation, reinforcing feelings of being different or damaged. Periods of hospitalisation could also amplify isolation, through enforced aloneness and disconnection from supports and society. Participants internalised the implied message that they were dangerous or needed to be contained away from society; leading to further isolation. This was described by one participant:

*“… so I felt like I could form no true friendships or relationships or anything like that. So I thought it’s just best if I isolate” – P11*.

## Discussion

The findings of this study provide new knowledge of how experiences of shame in people living with psychosis relate to past trauma. The narratives highlight the ways in which trauma, through shame, continues to impact on peoples’ lives through disconnection from self, others and a sense of aliveness. As recognition of the relationship of trauma to psychosis grows, and interests in shame also arise, these findings provide a detailed understanding of the nature and role of shame in the relationship between trauma and psychosis.

Participant’s accounts showed that shame can keep the effects of trauma present through ongoing feelings of differentness or defectiveness, or the seeming development of a ‘shame lens’ through which participants interpret the world. This aligns with findings in broader trauma research which suggest adverse experiences can influence the development of negative self- and other schemas (Horowitz, 1986), including shame-based self-schemas (Young et al., 2003). Lee et al. (2001) suggest that feelings of shame arise when meaning is given to an event that is congruent with underlying shame-based beliefs about the self. Once reactivated, these shame schemas serve as a filter through which the person perceives the world (Lee et al., 2001). In the current study, experiences of psychosis appear to reactivate shame self-schemas, providing further support for shame beliefs held about the self, developed through trauma. Schema therapy may be effective in reducing early maladaptive schemas, including shame-based schemas (Young et al., 2003), and preliminary support suggests that it may also reduce ongoing effects of trauma (Peeters et al., 2022). Future research could examine whether these findings can also be applied to people living with psychosis and additionally, whether this in turn influences experiences of psychosis.

In this study, the accounts provided suggested that efforts to manage shame associated with past trauma can lead to disconnection from the self. One explanation may be a disruption to narrative identity where exposure to adverse events has impeded the development of a coherent life story, and in turn sense of self (Grunfeld & Fulford, 2025). Narrative identity development could be further impacted as individuals avoid revisiting shame laden experiences and therefore miss opportunities to process and integrate these events into their broader life narrative. As research on narrative identity and psychosis continues to grow (Wiesepape et al., 2023), there may be future opportunities to explore shame’s role within this and ways in which it could be addressed.

Participants’ descriptions of neglecting or negating their own needs and experiences of rejecting of parts of their selves may align to processes associated with posttraumatic dissociation. Dissociation is noted as a “division of personality” and arises when an individual is not able to integrate adverse experiences (Nijenhuis & van der Hart, 2011). This may be a way of protecting an individual from intolerable experiences or emotions such as trauma, and has been strongly linked to shame (Dorahy, 2007). Increased feelings of shame are thought to increase dissociative experiences (Dorahy et al., 2017), with implications for the delivery of treatment and support. Professionals supporting people living with psychosis should be aware that dissociation may be perpetuated when feelings of shame are provoked, and be mindful of ways to reduce this in the delivery of care, for instance, through shame-sensitive practice (Dolezal & Gibson, 2022).

In addition to dissociation, other pathways for how shame may mediate the trauma-psychosis relationship were apparent across the accounts provided and align to the mechanisms proposed by McCarthy-Jones’ model (2017). Within this model, shame arising from past traumatic events may lead to the experience of hallucinations via coping responses including rumination, hypervigilance, dissociation and emotional suppression. The findings indicate that participants’ efforts to avoid activation of shame associated with trauma can lead to emotional suppression of past events and hypervigilance to future shaming. Participants accounts were often characterised by a reluctance to think about negative events and emotions whilst also being alert to, or in anticipation of further shame. Better understanding of how shame influences these paths is therefore of clinical importance. Research examining interventions that target each of these pathways and observing their impact is needed to further determine their strength and involvement.

The social defeat hypothesis offers an alternative mechanism through which shame could be considered to influence the relationship. Individuals are described as experiencing social defeat when they assume a subordinate position as a way of self-preservation, as is often the case in interpersonal trauma (Selten & Cantor-Graae, 2005; Troop & Hiskey, 2013). Social defeat has been linked to an increased likelihood of schizophrenia, acting as a mediator between a range of social adversities (Selten & Cantor-Graae, 2005). In this study, shame associated with holding a perceived lower social rank may have shaped negative views of the self that were then compounded by experiences of psychosis.

Similarly, within the Power, Threat, Meaning Framework (PTMF) shame is considered as both a potential threat arising from negative operations of power, and as a meaning applied to perceived threats (Johnstone et al., 2018). Support for both were observed in this study. For example, in participants’ descriptions of feelings of shame as a result of rejection or neglect (relational/social threat) and anticipation of this occurring again in the future; and in the ways participants described being ‘damaged’ as a result of past adversity. In this context, psychosis experiences such as hearing voices are viewed as a ‘threat response’ to manage the associated distress. Supporting individuals to explore the meanings they hold about events may aid in recognising where current feelings of shame arise from, and with appropriate psychological supports, encourage people to adopt new meanings that lead to less shame.

In addition to the support that these findings lend to theoretical models of the trauma and psychosis relationship, and shame’s mediatory role within this, the information about the role of shame, has implications for trauma-informed approaches to mental health care. Trauma-informed approaches to care recognise the influence that past adverse experiences may have on current behaviour and have been increasingly included in policy and practice. However, they have also been criticised for lacking clear application to practice (Isobel, 2021a). While the principles that guide practice to avoid re-traumatisation in services may naturally minimise triggering shame, shame is not explicitly addressed and greater recognition and understanding of its ongoing impact may inform service planning, treatment formulations and effective care delivery for people living with psychosis.

### Limitations

Discussing trauma and shame is known to be difficult, with feelings of shame potentially reactivated (Taylor, 2015). Although this study was conducted in a trauma-informed way, it is possible that participants did not share all aspects of their experiences, or their feelings about them, to protect themselves from distressing memories or emotions. Further, this study is an interpretative analysis undertaken by clinicians and academics. Whilst there was lived experience input into the study design and processes, and rigour and trustworthiness were actively attended throughout, there may be other ways to understand the relationship between trauma, shame and psychosis, including from the perspectives of people with lived experience.

## Conclusion

The findings from this study provide new information on how shame experiences in people living with psychosis are related to past trauma. The study found that shame may play a key role in maintaining the influence of trauma on sense of self and on the way in which people engage with the world, including in the context of psychosis. Future research could focus on shame interventions and whether addressing shame could alleviate some of the influence trauma has on people experiencing psychosis.

## Supplementary Information

Below is the link to the electronic supplementary material.

ESM 1 (DOCX 28.0 KB)

## Data Availability

Data from this study will not be shared to protect participant’s confidentiality. This is a qualitative study and thus the data set consists of transcripts containing sensitive information.
