# Supplementary material for: “I Just Feel Disconnected”: How Feelings of Shame Relate To Experiences of Trauma in People With Psychosis
Source: Community Ment Health J. 2025 Oct 2;62(2):316–24. doi: 10.1007/s10597-025-01525-1 (PMC12852282; doi:10.1007/s10597-025-01525-1)
Supplement: Supplementary file 1 — (DOCX 28.0 KB) [file 10597_2025_1525_MOESM1_ESM.docx]

**Introductory question**

Before we get into the formal questions, I’d like to ask a few questions to find out a bit more about you.

**Identity**

Firstly, I’m wondering what makes up you. So for example, some people may describe themselves as having different ‘parts’ or roles. What would you say are the main parts of your identity?

**Strengths**

- What would you say are the positives or strengths about you?
- Do you think this is something that has always been the case or have you developed these following mental health difficulties?

**Experiences of psychosis**

Thank you for sharing, and letting me get to know you a bit better. If it’s ok with you, I’d like to ask you now about your mental health experiences.

If there are any you would prefer not to discuss, let me know and we can skip ahead.

| **Experiences of psychosis** |
| --- |
| **What was the health problem or difficulty that led to you being in contact with mental health services?**  Follow-up questions   - *Why do you think they started at that time?* - *What do you think caused them?* |
| **What has that been like for you**?  *Prompts: How has it affected/does it affect your life?* |
| **How have these mental health experiences made you feel about yourself?**  *Prompt: For instance, some people who have these types of mental health experiences say they make them feel as though they have grown as a person, whereas others describe feeling small or as though something is missing in them.*  Follow up questions   - *Where do you think these feelings come from?* - *What do you do to manage these feelings?* |
| **Are any of your mental health experiences you’ve described related to past events or memories?**  *Prompt: If so, are you able to tell me how?* |
| **Would you be able to tell me about any strategies you use to manage the psychosis experiences you’ve described?** |
| **What do you think of the term ‘psychosis’?**  *Prompt: Is there another term you like to use to describe your mental health?* |
| **What do you think are some of the challenges people who experience [psychosis] face? Has this been the case for you?** |
| **Others have mentioned a sense of shame around their mental health experiences, is this something you can relate to?** |

**Emotions**

(indications: feeling of defectiveness/being damaged, feeling judgement from others, feeling less than others, worthlessness, coping mechanisms involve hiding, withdrawal, isolation or anger/blaming others)

| **Emotions** |
| --- |
| I noticed on the questionnaire that you mentioned that ___________________________ __________________________________________________________________________________________________________________________________________________ **where do you think those feelings come from? Are they related to only the psychosis experienced or have you felt like that before?** |
| **Would you be able to tell me more about what happened when you felt (shame term used)?**  Follow up questions  What was happening at the time? |
| **Where do you think that feeling comes from?** |
| **Would you be able to tell me what you do when you have those feelings of shame?**  *Prompt: For example, some people may try to block out thinking about any events that were embarrassing, others may not be able to stop thinking about it?* |

Thank you for answering these questions, I appreciate they can be difficult. Are you ok to continue or would you like a break? (ongoing consent check)

**Life events**

It’s common for people who have experienced psychosis to have lived through some difficult life events (if ask for clarification - things such as bullying, neglect from carers, or physical or sexual abuse.)

Would it be ok to ask you a few questions about this? If you feel like any of the questions are too confronting and would like to take a break, please let me know. Or if you would prefer to skip over any we can do that too.

****If ok to continue****

I just want to remind you that anything we talk about in the interview is confidential. I also want to let you know that I will not be asking for specific details about any difficult events, as this is best discussed in a therapeutic setting. If you feel you need more support to discuss this further, I can direct you, or your mental health team to appropriate resources.

**Early experiences**

So, I will start by asking some questions about your early memories and what these meant for you. Please let me know if there are any you’d prefer not to answer.

| **Early experiences** |
| --- |
| Would you be able to tell me what your early life was like?  *Prompts: What made it that way? Can you remember any specific sounds or smells? How did that make you feel at the time?* |

**Life Events**

| **Life events** |
| --- |
| **How do you think your early life has influenced the person you are today?**  Follow up question  In what ways do you think it has influenced you? |
| **Have there been any events in your life that you would consider traumatic?**  *Prompts: For instance, where you were harmed or felt at risk of harm?*  ***If no skip to ***** |
| **What impact do you think this has had on you?** |
| **What did these experiences mean for you?**  *Prompt: For instance, what did you believe about yourself at the time?* |
| **Often, people feel strong emotions after a difficult life event, Is this true for you?**  *Follow up questions:*  Would you say this still affects you now? *Are there certain situations that might trigger these particular feelings?* |
| **Do you think this event has influenced your mental health experiences of [psychosis]?**  *Follow up question:*  *In what ways?* |
| ****Some people describe their [psychosis] experiences as traumatic. For example, hearing distressing voices. Has this been the case for you?**  *Prompt: (if yes) what is it about this experience that you found distressing?* |
| **Sometimes people describe feeling upset about the things they did, or the way they behaved when they were experiencing psychosis. Would this be true for you?**  *Prompt: (if yes) would you be comfortable to tell me more about this?* |
| **Have you previously shared this/these events with others around you?**  *Prompts: how have they reacted when you have told them about it? How did this feel for you?*  *Prompts (if not): what do you think has prevented you from doing so?* |
| **Have you ever been asked about difficult life events by mental health professionals?**  *Yes – How did this feel? No – Would you have preferred to have been asked?* |
| **Have you received any support for the difficult life events you’ve mentioned?**  *Follow up question:*  *Do you think this was/would have been helpful?* |

**I just wanted to check if there's any other questions, any other thoughts you have about life events that I haven't asked about that you thought I would, or that you wanted to share?**

Thank you for sharing this part of your life with me, I appreciate that it can be hard. Would you like a break?

The last few questions are about your personal experiences of mental health services and the support you may have received.

I’d just like to remind you that your interview is confidential, and nothing you tell me will be relayed back to any service and will not affect the supports you receive in any way. But if there are any questions, you’re not comfortable answering, that’s ok we can move on.

**Service engagement**

| **Episodic** |
| --- |
| **Overall, what have your experiences with mental health services been like?**  *Prompts: What made them good/bad? Is there any particular experience you’re thinking of?* |
| **Do you think services play a role in reducing shame?**  *Prompts: Are they doing a good job of doing this? Is there anything they could do differently?* |
| **Is there anything services could have done that might have made your experience more positive?**  *Prompt*: *What might have helped you to feel safer?* |

**If shame mentioned in response to questions about services:**

- You mentioned that [your contact with services has led to feelings of shame]. Would you be able to tell me more about this?
- What would have helped to reduce the feelings of shame? Is there anything they could have done differently?

**Summary**

Summarise key parts of interview.

Thinking back on all we’ve discussed, is there anything else you’d like to talk about, or anything you think I’ve missed that is an important part of your story?

**Concluding interview**

Do you have any questions?

Thank you for your time today and for sharing your experiences with me. It’s important to me, that I represent the things you have told me accurately, and the recording of this interview will be transcribed to assist with this. Some people may wish to read a summary of this interview and add any further thoughts, would this be of interest to you?

**Feedback on interview questions**

As we seek to better understand the experience of psychosis and difficult emotions such as shame, it’s important to us that we’re asking the right questions and not unnecessarily causing distress. If you have any feedback on the questions we’ve asked or think there are other questions that should be included, please let us know.

**Thank participant for time and arrange follow-up check-in call.**
